# Supplementary material for: Metabolic, Hematological, and Functional Health in Adults with Down Syndrome and Significance of Parental Health Literacy: A Cross-Sectional Study
Source: Healthcare (Basel). 2025 May 21;13(10):1212. doi: 10.3390/healthcare13101212 (PMC12111464; doi:10.3390/healthcare13101212)
Supplement: Supplementary file 1 [file healthcare-13-01212-s001.zip › healthcare-3637029-supplementary.pdf]

Supplemenary Table S1. Descriptive statistics and gender differences in all study variables.

Variable	Total sample (n=18)		Female (n=11)		Male (n=7)		T-test	
	Mean	SD	Mean	SD	Mean	SD	t-value	p
Age	28.22	7.67	28.64	9.01	27.57	5.50	0.28	0.78
BH	153.42	8.67	147.55	4.18	161.50	6.28	-5.83	0.00
BM	67.93	16.66	65.60	18.06	71.14	15.09	-0.71	0.49
BMI	29.52	7.26	30.12	8.15	28.59	6.07	0.43	0.68
FM%	23.93	12.03	28.62	11.43	16.57	9.43	2.32	0.03
MM (%)	43.07	6.80	40.41	6.45	47.24	5.34	-2.33	0.03
Glucose(g/dl)	5.69	0.52	5.52	0.51	5.94	0.45	-1.86	0.08
Hemoglobin (g/dl)	15.41	1.05	14.99	1.00	15.99	0.86	-2.27	0.04
Hematokrit (L/L)	45.62	3.58	43.88	3.24	48.00	2.58	-2.97	0.01
TCHOL (mg/Dl)	148.05	33.20	156.55	35.03	136.38	28.51	1.34	0.20
HDL (mg/dL)	32.89	11.90	39.55	9.86	23.75	7.74	3.76	0.00
TRIGL (mg/dL)	109.00	35.60	104.73	38.74	114.88	32.35	-0.60	0.55
LDL (mg/dL)	93.74	30.38	102.73	20.19	81.38	38.58	1.57	0.13
CHOl/HDL	4.64	2.08	4.15	1.06	5.33	2.93	-1.24	0.23
nonHDL (mg/dL)	115.16	29.95	117.00	33.64	112.63	26.03	0.31	0.76
6MWT (m)	513.84	89.77	470.09	91.74	574.00	39.44	-2.99	0.01

Note: BH – body height; BM – body mass; BMI – body mass index; FM% - fat mass percentage; MM% - muscle mass percentage; TCHOL - total cholesterol; HDL - high-density lipoprotein; TRIGL - triglycerides; LDL - low-density lipoprotein; CHOL/HDL - cholesterol to HDL ratio; nonHDL - non-HDL cholesterol; 6MWT - six-minute walk test
